# Why mothers die at a busy tertiary urban hospital in Kampala, Uganda: a comprehensive review of maternal deaths 2016–2018 and implications for quality improvement to reduce deaths

**DOI:** 10.4314/ahs.v22i2.57

**Published:** 2022-06

**Authors:** Imelda Namagembe, Noah Kiwanuka, Josaphat K Byamugisha, Sam Ononge, Jolly Beyeza-Kashesya, Dan K Kaye, Ashley Moffett, Catherine E Aiken, Annettee Nakimuli

**Affiliations:** 1 Department of Obstetrics and Gynaecology, School of Medicine, Makerere University College of Health Sciences, Uganda, P.O.Box 7072, Kampala, Uganda; 2 Mulago National Referral and Teaching Hospital, P.O.Box 7051, Kampala, Uganda; 3 Department of Epidemiology and Biostatistics, School of Public Health, Makerere University College of Health Sciences, Uganda, P.O.Box 7072, Kampala, Uganda; 4 Department of Pathology and Centre for Trophoblast Research, University of Cambridge, Cambridge, United Kingdom; 5 Department of Obstetrics and Gynaecology, University of Cambridge, Box 223, The Rosie Hospital and NIHR Cambridge Biomedical Research Centre, Cambridge CB2 0SW, United Kingdom

**Keywords:** Maternal-deaths, death-review, preventability, quality improvement

## Abstract

**Background:**

Reviewing maternal deaths and drawing out lessons for clinical practice is part of an effective cohesive intervention strategy to reduce future deaths.

**Objective:**

To review maternal deaths at the National Referral hospital in Kampala over a 3-year period (2016–2018) to determine causes of death, extent of preventability, proportion of deaths notified and audited as per national guidelines.

**Methods:**

Trained-multidisciplinary panels (obstetricians and senior midwives) conducted retrospective reviews of maternal deaths that occurred.

**Results:**

Major causes of deaths: obstetric haemorrhage (158/350; 45%), hypertensive disorders of pregnancy (87/350; 25%) and infection (95/350; 27%). Overall, 294/350 (84%) of maternal deaths were considered preventable. In 95% (332/350) of cases, delays within healthcare facilities were identified (64%; 226/350). We note that only 115/350 (33%) cases had been audited. This proportion did not change during the studied period. In 48% (167/350) of cases, notification to the Ministry of Health occurred, but only 11% of deaths (39/350) were notified within the recommended 24-hours.

**Conclusions:**

A high proportion (84%) of deaths were preventable. Significant delays to care occurred within health-care facilities. Results suggest that a well-supported, and timely maternal death review process with targeted and pragmatic interventions might be effective in reducing maternal deaths in this setting.

## Introduction

Maternal deaths continue to occur at unacceptably high rates globally, with more than two-thirds of all maternal deaths occurring in sub-Saharan Africa 1.The United Nation's Sustainable Development Goal 3.1 includes the aim of reducing the global maternal mortality ratio (MMR) to <70 per 100,000 live births by 2030 2, however many low resource obstetric settings are still some way off this target 3.

It is estimated that as many as 1 in 50 maternal deaths worldwide occur in Uganda 4. In 2015, UNICEF set a country-specific MMR target of 211 per 100,000 livebirths by 2017 for Uganda 5. However in 2016, Ugandan estimates suggested an MMR of ∼336 per 100,000 livebirths 6. This represents an improvement from 438 per 100,000 livebirths in the decade between 2006–2016. However, Uganda is clearly some way off meeting the minimum national MMR target of <140 per 100,000 livebirths by 2030 7, 8.

Identifying context-appropriate and feasible interventions to prevent maternal death is essential to reduce the MMR. A key part of understanding what interventions are likely to be effective in preventing future deaths is to conduct full reviews of maternal deaths to establish causality and preventability. Globally, implementing robust and comprehensive systems of maternal death review has been shown to be a key element of cohesive strategies to reduce maternal mortality 9, 10.

Previous work suggests that the majority of maternal deaths in Uganda are from potentially preventable causes such as haemorrhage, hypertensive disorders, and sepsis^11^–^13^. Assessing and understanding preventability is a key element of maternal death review, particularly in contexts where the MMR remains high. A high proportion of preventable deaths suggests that appropriately-targeted interventions are likely to be effective in reducing MMR. The key aim of our study was, therefore, to describe the major causes of maternal death at Mulago National Referral Hospital, a very busy urban service in Kampala, in order to assess preventability.

In Kampala, there is a mixed health system comprised of both private and public, but predominantly private sector. There is no health insurance for most people and the urban poor use the few functional public health facilities available 14, 15. The national policy recommending implementation of maternal death reviews was first introduced in 1998. In the intervening decades there have been other policy interventions, including a Presidential directive in 2008, aimed at improving the number and quality of reviews nationally. Despite these efforts however, there remains no clear understanding of what proportion of maternal deaths are subjected to a formal death review with resultant potential learning and opportunities for clinical improvement at the National Referral Hospital. This tertiary facility is the major training site for Makerere University medical students and others. Therefore, second aim of our study was to systematically assess the position of maternal death review and reporting at the National Referral Hospital.

## Methods

We conducted comprehensive retrospective case reviews of all maternal deaths that occurred at the study center during the 3-year study period (1^st^ January 2016 – 31^st^ December 2018). The study setting was the Department of Obstetrics and Gynaecology of Mulago National Referral and Teaching Hospital. The department was originally located on Mulago Hill but temporarily transferred to Kawempe in August 2016 to accommodate renovations at the Mulago Hill site. The center is one of sub-Saharan Africa's busiest maternity units and accepts referrals from all over Uganda in addition to serving a local low-resource urban population. The number of deliveries annually ranged from 23,000 to 27,000 during the 3-year period. On average the study center handles ∼70 deliveries per day, including 20–25 emergency Caesarean sections^16^. The average number of maternal deaths at the center every year is ∼136–140 13.

There were 401 maternal deaths identified in the study center's register of admissions, in which the outcome of every admission of a pregnant woman was recorded. Registry records were cross-referenced against mortuary records for all women of reproductive age. Maternal death was defined as the death of a woman during pregnancy or within 42 days of pregnancy ending from any cause. The full case records and any other available documentation for all identified maternal deaths were manually retrieved by records officers who received study-specific training. Key contemporaneous medical records, any evidence of previous maternal death review or audit using Ugandan Ministry of Health maternal death audit / review form plus notification forms. Fifty-one cases were excluded (34 records could not be traced, 15 records were incomplete, and 2 deaths occurred >42 days after the end of pregnancy). Thus, 350 maternal deaths were included in the analytic sample.

Available maternal death records were anonymized by the trained research midwives prior to confidential review by the study team. Blocked name of woman who died, any named relatives, and all healthcare staff. Doctors were identified by grade and other staff by role, e.g. ‘Midwife 1’. All personnel involved in handling or reviewing cases signed a confidentiality statement (adapted with permission from MBRRACE-UK 17. Each case file was reviewed in full by a multidisciplinary panel that included at least one obstetrician and one experienced midwife who were familiar with the study setting, including detailed knowledge of local clinical practice, guidelines, and resources. All health-care professionals involved in the case review panels received study-specific training in maternal death review. In total, 20 panel members were trained (12 fully-certified obstetricians and 8 midwives each with >10 years of clinical experience). Additional opinions were sought by review panels from other local experts such as anesthesiologists, physicians from various specialties, and neurosurgeons where appropriate. A total of 350 case summaries was produced. This included a structured collation of key demographic, obstetric, and clinical management information, in addition to a detailed narrative summary of the events leading to death. The panel also recorded their view on the most likely major contributing factors to death, the preventability of each death, and noted any significant delays or missed opportunities in care. Delays in care included any delays in initiating care at each step of the patient's journey, and significant delays thereafter, for example awaiting space in an operating room.

Preventability was defined as the chance of saving the life of the mother within the existing health system in Uganda based on the documented trajectory of events until her death, and was assessed by each panel in light of their expertise and experience. The question was answered after studying the documented events: ‘Was this a preventable maternal death? Possible responses were ‘Yes/No/Difficult to determine’. This was followed by: ‘What was the chance of preventing such a death? Options were: ‘Good Chance/No Chance/Difficult to determine’. As the study was conducted at the national tertiary referral center for obstetrics, no higher-level medical facilities were available. A referred patient was any mother who had documentation that any medical management took place prior to arriving at the study centre. Each case summary was further independently reviewed by an international obstetrician, who made several visits to the study site to become familiar with resources and organizational factors within the setting. The international obstetrician has previous experience with confidential enquires into maternal death in other settings. She provided an external unbiased opinion of the review process but did not influence the classification of the review.

Ugandan national policy specifies that all maternal deaths should be notified to the Ministry of Health within 24 hours of the death occurring, using a one-page proforma^18^. A copy of this form is retained within the records office. Where such a form was present, the death was classified as ‘notified’ and the date of notification recorded. Any evidence of a local audit process was also sought and the date recorded. This information was used to construct a timeline of notification and review for each case. Case summaries from the review panels were checked for completeness by the study team and returned to the next meeting of the panel if any data were missing, incomplete, or ambiguous. The retrospective data was complete in regards to the socio-demographic variables (age, marital status, referral status) and clinical information (Table 1). However, there was commonly missing information on the antenatal care the mothers received, particularly for mothers who were transferred in from other obstetrics settings. Trained data clerks input the data from the case summaries into EpiData version 3.1 using a double-entry system to enhance the consistency of data capture. Analyses were performed using Stata, Version 12 19 and R version 3.6.3 20. Findings were considered statistically significant at an alpha level of 0.05.

**Table 1:** Demographic details of women whose cases were included in the maternal death review

Characteristic		Mean (±SD) or Number (%)
Maternal age (years)		26.7 (± 6.3)
	< 20	50 (14.3%)
	20–24	91 (26.0%)
	25–29	91 (26.0%)
	30–34	70 (20.0%)
	≥ 35	47 (13.4%)
	*	01 (0.3%)
Missing		

Parity	0	60 (17%)
	1	87 (25%)
	2–4	162 (46%)
	≥5	35 (10%)
	Unknown	6 (2%)

Marital status	Married	221 (63%)
	Single/divorced/widowed	13 (4%)
	Unknown	116 (33%)

HIV status	Positive	49 (14%)
	Negative	94 (27%)
	Not tested	207 (59%)

Estimated distance of residence from study center (km)		36.1 (± 59)

Referral status	From other healthcare facility	238 (68%)
	Not referred (Local patient)	112 (32%)

Timing of death	Prior to 28 weeks	51 (15%)
	Antenatal >28 weeks	33 (10%)
	Intrapartum	138 (39%)
	Postpartum	126 (36%)

The study was approved by the Makerere University School of Medicine Higher Degrees Research and Ethics Committee (SOMREC; #REC Ref 2018-001) and by the Uganda National Council for Science and Technology (UNCST; Ref SS4797).

## Results

### (i) Demographics and cause of death

The women who died were aged between 14–47 years (Table 1). The median parity was 2, with a range of 0–13 previous births. It is estimated that maternal deaths included in this review left 924 children motherless. The majority of women were married (63%, 221/350) and 32% (122/350) of women who died were not referrals, whereas 68% were referred from other healthcare settings. The proportion of HIV positive status in the maternal death cases was higher than previously reported for pregnant women in Uganda 21 (14%, 49/350), but the HIV status of the majority of women who died was unknown (207/350, 59%).

The mean distance travelled to reach the study center was 36km (median 13.5km, range 0.6–433km). For local women admitted directly to the study center the mean distance travelled was 17.7km, whereas for women referred from other facilities it was 44.8km. Intrapartum deaths (138/350; 39%) and postpartum deaths (126/350; 36%) accounted for the majority of cases.

The most common causes of death were obstetric hemorrhage (45%), hypertensive disorders of pregnancy (28%), and infection (27%) (Table 2). Infection was further sub-divided into puerperal sepsis (13%) and other maternal infections (23%). Other causes of death each accounted for <10% of cases (Table 2).

**Table 2:** Causes of maternal death. Deaths could be attributed to more than one major cause

		Audited	Notified	Preventable
Cause	Total	N=115	N=167	N=294
	N=350	(32.9%)	(47.7%)	(84.0%)
Obstetric hemorrhage	158 (45%)	53 (34%)	81 (51%)	151 (96%)*
Hypertensive disorders of pregnancy	87 (25%)	30 (35%)	37 (43%)	72 (83%)
Puerperal sepsis	54 (15%)	17 (32%)	23 (43%)	48 (89%)
Other maternal infection (e.g. malaria, HIV/AIDs, TB)	41 (12%)	10 (24%)	12 (51%)	26 (63%)*
Maternal medical conditions (e.g. diabetes, cardiac disease)	33 (9%)	11(33%)	16 (49%)	16 (48.5%)***
Spontaneous miscarriage/ abortion-related deaths	20 (6%)	7 (35%)	7 (35%)	16 (80%)
Anesthesia-related deaths	17 (5%)	10 (59%)*	7 (41%)	13 (76.5%)
Ectopic pregnancy/ gestational trophoblastic disease	7 (2%)	5 (71%)*	2 (29%)	5 (71.4%)

(e.g. obstetric haemorrhage plus puerperal sepsis). The number and percentage of deaths from each cause that were audited, notified, and classified as preventable respectively are shown. Multivariate logistic regression models were used to determine causes of death that were more likely than others to be audited, notified, or classified as preventable respectively. Deaths due to anaesthesia-related causes and due to ectopic/trophoblastic disease were significantly more likely than other deaths to be audited. Deaths due to haemorrhage were significantly more likely than those due to other causes to be classified as preventable, whereas those due to maternal infectious disease or medical conditions were less likely to be classified as preventable.

* p<0.05

*** p<0.001

As the study center is a national referral center, a subgroup analysis was performed comparing the distribution of causes of maternal death in the women who attended because the study center was their local hospital (n=112) to those who were referred from other healthcare facilities (n=238). There was no significant difference between the local and referred cases in the timing of deaths relative to delivery (postpartum deaths 36% v. 37%; p=0.78). Obstetric haemorrhage and hypertensive disorders were significantly more common causes of death in women who were referred from other facilities (Table 3), whereas puerperal sepsis was more common in local women. However, the top 3 major contributing factors to maternal death were the same in the local compared to the referred population.

**Table 3:** Distribution of major contributing factors to maternal death by referral status. Maternal deaths could be attributed to one or more major factor. Chi-squared tests were used to compare the likelihood of each cause of death in the referred versus the local population

		Local (Not	
Major contributing factors	Referred	referred)	p value
	N=238 (68%)	N=112 (32%)	
Obstetric hemorrhage	112 (48%)	41 (37%)	0.028
Hypertensive disorders of pregnancy	69 (29%)	18 (16%)	0.009
Puerperal sepsis	29 (12%)	25 (22%)	0.014
Maternal infectious disease	23 (10%)	18 (16%)	0.082
Maternal medical conditions	22 (9%)	11 (10%)	0.863
Spontaneous miscarriage/ abortion-related deaths	10 (4%)	10 (9%)	0.074
Anesthesia-related deaths	13 (5%)	4 (4%)	0.442
Ectopic pregnancy/ gestational trophoblastic disease	5 (2%)	2 (2%)	0.844

### (ii) Delays in care

At least one delay in care that contributed to death was identified in 95% (332/350) of the case reviews. In 58% (203/350) there were delays at more than one point that may have contributed to adverse outcomes. The most common point of delay was at the study center itself, where a significant delay in care was identified in 64% (226/350) of cases (Table 4). Delays in care at the study centre included initiating and continuing appropriate care such as delay in performing a caesarean section, lack of /inadequate blood products and supplies, inadequate monitoring of patients (intra-partum, post-delivery, intra-operative or post-operative period), and lack of critical human resource.

**Table 4:** Delays in care identified in maternal death cases, classified by the point at which delay occurred

	Frequency (%)
Type of delay in accessing care	N=350
Delay at study center	226 (64%)
Delay at individual or family level	131 (37%)
Delay at other healthcare facility (government-run)	108 (31%)
Delay at other healthcare facility (private)	90 (26%)
Delay in transport from home	13 (4%)
Delay with traditional birth attendant	07 (2% )

### (iii) Death review process and preventability

**Figure 1:**
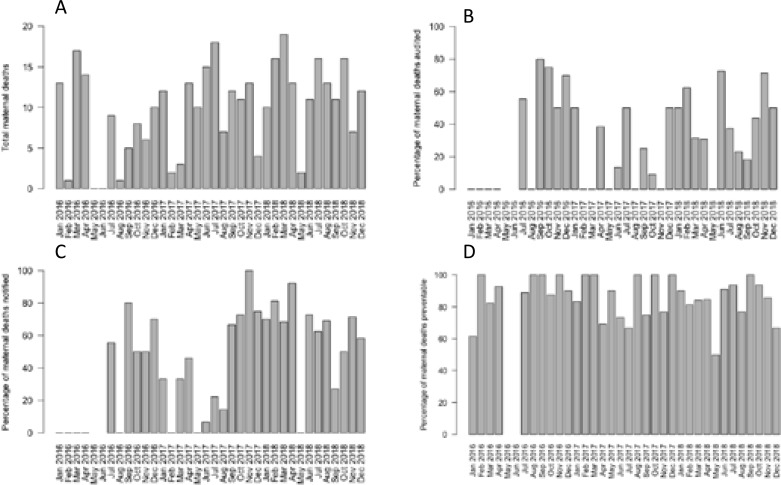
Maternal deaths 2016–2018 by month. A) Total deaths, B) Percentage of maternal deaths that were audited, C) Percentage of maternal deaths that were notified, D) Percentage of maternal deaths that were considered preventable on review.

The total number of maternal deaths per month showed no significant difference across the 3-year study period, ranging from 0–19 (Figure 1A). During the latter half of 2016, the study center changed in location within urban Kampala, and some loss of information during this move may account for low numbers of deaths recorded in some months (May/June 2016).

Evidence of local audit was present in 33% (115/350) of case records. The percentage of deaths audited per month was unchanged across the study period, ranging from 0–80% (Fig 1B). The median time to audit was 132 days (range 0–389) and 10% (34/350) of cases were audited within 1 month of death. Deaths due to anaesthetic causes (p=0.02) or ectopic pregnancy/gestational trophoblastic disease (p=0.04) were significantly more likely to be audited than deaths from other causes (Table 2), although the absolute numbers of these deaths were very small. There was no significant difference in the percentage of deaths audited between the local population and those referred from other facilities (29% v. 34%, p=0.35).

Evidence of notification to the Ministry of Health was present in 48% (167/350) of the case records. The percentage of deaths notified per month did increase significantly during the study period (Fig 1C), however the average percentage during the final 6 months of the study (Jun–Dec 2018) remained stable at 56%. The median time to notification was 126 days (range 0–389) and 11% of deaths (39/350) were notified within the recommended 24-hour time-frame. There were no significant differences in the likelihood of notification between different causes of death. There was no significant difference in the percentage of deaths notified between the local population and those referred from other facilities (51% v. 55%, p=0.43).

The review panels considered that 84% (294/350) of deaths were preventable within the Ugandan health system, taking into account local contextual factors. In 3 cases (1%) the death was assessed unpreventable, and in a further 53 cases (16%) preventability could not be determined. The percentage of deaths that were preventable per month was unchanged across the study period, ranging from 50–100% (Fig 1D). Of the potentially preventable deaths, the panel considered that there was a good chance of preventing 55% (161/294) of deaths with some chance of preventing the remainder (133/294, 45%). Deaths from obstetric haemorrhage were significantly more likely than average to be preventable (p<0.05), whereas deaths caused by other maternal infections (p<0.05) and deaths caused by maternal medical conditions (p<0.001) were significantly less likely to be classified as preventable (Table 2). There was no significant difference in the percentage of deaths considered preventable between the local population and those referred from other facilities (86% v. 83%, p=0.62).

## Discussion

In this low-resource urban setting, the major causes of maternal mortality over a three-year period were obstetric haemorrhage, hypertensive disorders of pregnancy, and puerperal sepsis. These causes together accounted for, or were major contributors to, nearly 80% of all maternal deaths. Although the relative proportions of these three causes varied somewhat between women who were referred for escalating care compared to the local population for whom the study center provided first-line care, they still accounted for the majority of deaths in both groups. The proportion of all deaths that were preventable was high in this population and did not reduce over the 3-year study period. Maternal death review remains at low levels overall, with only 33% of cases undergoing local audit. Notification of maternal deaths to the Ministry of Health did increase over the 3-year study period, with over half of all deaths (56%) reported during the last 6 months of the study.

Our results suggest that maternal deaths at the study centre occurred primarily from a small number of preventable causes. Our optimism that effective interventions to prevent maternal death would be feasible in this setting is reinforced by the high number of deaths in which delays contributing to death occurred at the study center rather than during other parts of the pathway. Delays at the study center are much more likely to be readily modifiable than delays prior to arrival, which are more likely to be multifactorial and outside the scope of direct healthcare intervention. It is therefore possible that highly targeted interventions could be identified and potentially have a major impact on the number of women dying overall.

The demographic profile of the women who died in Kampala was similar to that described in other African countries, which also report high maternal mortality in young parous women 22, 23. The causes of maternal death in this setting are similar to those identified in other low-resource obstetric settings 24–26 and in previous studies in urban Uganda 13. Haemorrhage was not only the most frequent contributor to death in our study but also the cause most often assessed as preventable. This is comparable to data from the national Ugandan maternal mortality 27, financial year 2017/2018), where obstetric haemorrhage accounted for (35.5%) of the 1,111 maternal deaths, followed by hypertensive disorders (12.5%), sepsis (8.9%), abortion (5.3%) and ectopic gestation (1.1%). The maternal death notification and review at national level were both at 49% which is the same as our study and considerably less than the annual target of (70%) in the Health Sector Development Plan (HSDP) plan 27.

Obstetric haemorrhage is a major cause of maternal death globally 28, 29, but several trials have shown promising results for interventions suitable for implementation in low-resource settings to reduce haemorrhage-related deaths. These include uterotonics such as heat-stable carbetocin 30, although application in this setting may be limited by cost. Administration of tranexamic acid 31, and increased multi-disciplinary training in obstetric emergency drills 32 are also evidence-based interventions that may be suitable for implementation.

The potential for reducing maternal death with targeted interventions highlights the urgency of increasing the proportion of maternal deaths that are reviewed. The Ugandan Ministry of Health and international partners have continued to promote maternal death review with a particular emphasis on defining appropriate interventions to apply in clinical practice. In 2016, Uganda published a national strategy document that included a target of 70% of all maternal deaths undergoing local audit4. However, the proportion of deaths with any evidence of local audit in our study was considerably below this threshold and did not increase during the study period. There is an urgent need to understand why rates of local maternal death audit remain low in Uganda, despite national initiatives such as increased training in maternal death review. However, there is evidence from other African settings that even review of a relatively small percentage of deaths can result in the development of effective interventions to reduce the rate of maternal death 33.

An important finding emerging from our results is that there were no significant differences in the rates of the major causes of maternal death in the audited versus non-audited cases. This implies that there is not likely to be significant bias in the selection of cases that do undergo local review, however, more work is needed to understand why review is undertaken for some cases but not others. The results of our study highlight the urgent need for in-depth qualitative research involving healthcare professionals to understand the contextual barriers that need to be overcome to increase the proportion of maternal deaths that undergo timely review.

By contrast notification of maternal deaths to the Ministry of Health did increase during the study period, and was significantly higher throughout than the 22% reported nationally in 2016 4. This is a positive step towards robust epidemiological tracking of maternal deaths nationally. Optimising resource allocation and intervention is only possible on a background of good quality data, and it was encouraging to see positive steps towards this. A key advantage of the study design is that a large number of maternal deaths were reviewed by specifically trained review panels, all of which were multi-disciplinary. The study was conducted in a very high-volume center, which has one of the highest annual delivery rates in Africa. Moreover, the study center accommodates both a local low-resource urban population and tertiary-level referrals, allowing analysis that provides important insights into relative proportion of each cause of death in each group. Although this is a single center study, the inclusion of maternal deaths whose care began in various different regions across Uganda is an important advantage.

Study limitations include the incompleteness of documentation in several cases, for example with regard to antenatal care. In this setting there is no electronic capture of patients' information, and where women were referred from other healthcare facilities and regions there was frequently incomplete documentation of their care prior to transfer. However, majority of cases, particularly local women, included sufficient information to form a complete narrative summary. A further limitation was that post-mortem examinations are extremely rare in this setting, due to a variety of factors including resourcing and relatives declining post-mortem examinations. In a number of cases corroboration of the contemporaneous clinical impressions with pathology findings would have potentially altered the most likely cause of death 34.

## Conclusions

The majority of women whose deaths were included in this study were in their twenties, and the cohort were mothers to almost 1,000 young children. The impact of these tragedies on an individual, family, and community level is inestimable. However, maternal death review in this setting still remains considerably below target levels and hence urgent further work is required to understand how we can derive learning points and interventions from more of these deaths.

In this high-volume, low-resource obstetric setting, the majority of the deaths were preventable and subject to delay occurring within the medical setting. A few major causes accounted for the majority of deaths; obstetric haemorrhage, hypertensive disorders of pregnancy, and puerperal sepsis. Our results thus suggest that highly targeted interventions could be identified and may be effective in considerably reducing maternal deaths in this setting.

## Declarations

### Availability of data and materials

Due to the sensitive nature of the data processed in this study, the raw data will not be publicly available, but may be available from the corresponding author on reasonable request.
